# Improving HybrID: How to best combine indirect and direct encoding in evolutionary algorithms

**DOI:** 10.1371/journal.pone.0174635

**Published:** 2017-03-23

**Authors:** Lucas Helms, Jeff Clune

**Affiliations:** Evolving Artificial Intelligence Laboratory, University of Wyoming, Laramie, Wyoming, United States of America; Nankai University, CHINA

## Abstract

Many challenging engineering problems are regular, meaning solutions to one part of a problem can be reused to solve other parts. Evolutionary algorithms with *indirect encoding* perform better on regular problems because they reuse genomic information to create regular phenotypes. However, on problems that are mostly regular, but contain some irregularities, which describes most real-world problems, indirect encodings struggle to handle the irregularities, hurting performance. Direct encodings are better at producing irregular phenotypes, but cannot exploit regularity. An algorithm called HybrID combines the best of both: it first evolves with indirect encoding to exploit problem regularity, then switches to direct encoding to handle problem irregularity. While HybrID has been shown to outperform both indirect and direct encoding, its initial implementation required the manual specification of when to switch from indirect to direct encoding. In this paper, we test two new methods to improve HybrID by eliminating the need to manually specify this parameter. *Auto-Switch-HybrID* automatically switches from indirect to direct encoding when fitness stagnates. *Offset-HybrID* simultaneously evolves an indirect encoding with directly encoded offsets, eliminating the need to switch. We compare the original HybrID to these alternatives on three different problems with adjustable regularity. The results show that both Auto-Switch-HybrID and Offset-HybrID outperform the original HybrID on different types of problems, and thus offer more tools for researchers to solve challenging problems. The Offset-HybrID algorithm is particularly interesting because it suggests a path forward for automatically and simultaneously combining the best traits of indirect and direct encoding.

## Introduction

Evolutionary algorithms (EAs) automatically search a space of possible solutions to return high-performing solutions [1]. They routinely produce novel, effective solutions to many challenging problems, and often outperform human engineers [2–12]. One important domain is evolving *artificial neural networks* (ANNs), which are computational models inspired by the information processing capabilities of natural brains [1]. ANNs are typically complicated to construct by hand, but are capable of solving difficult computational problems, including object and symbol recognition for computer vision tasks and locomotion control for robots [13–15]. EAs can optimize the weights and architecture of ANNs, and have successfully designed ANNs for a variety of applications including robot controllers [4, 6, 15–18] and pattern recognition [5, 19–21].

The design principle of *regularity* is key to the success of EAs on regular problems [22]. Regularity refers to the compressibility of the information describing a structure, and typically involves symmetries and the repetition of design themes, with and without variation [23, 24]. Many natural organisms exhibit regularity through left-right symmetry and repetition of design motifs resulting from the reuse of genetic information when producing a phenotype, which allows for complex phenotypes to be described by compact genomes.

In EAs, the *encoding* describes how information is stored in a genome and converted into a phenotype [22]. EAs with *directly encoded* genomes, where each element of the genome encodes a separate aspect of the phenotype, are unable to exploit regularities in the problem because a solution discovered for one part of the problem cannot be reused to solve another part [22, 24, 25]. EAs with *indirectly encoded* genomes, where individual genome elements may encode for many phenotypic elements, are well suited to solve regular problems because they can reuse genetic information to create regular phenotypes [20]. Unlike direct encodings, indirect encodings can benefit from spatial organization of problem elements when the indirect encoding is a function of the geometric relationships between elements [19, 20, 26–29]. Mutations are also more likely to be advantageous to an indirect encoding than to a direct encoding since the effects of mutation on the indirectly encoded phenotype are coordinated and regular [15, 24, 25, 30]. For example, mutating an indirectly encoded ANN quadruped robot controller may have a coordinated impact on the gait properties for all legs, while a mutation to a directly encoded ANN controller is unlikely to do so. Previous work has shown that indirectly encoded solutions are more generalizable and more easily transferred to new tasks than directly encoded solutions [28, 29], which is useful for applications such as robotics, where the transfer of knowledge between tasks is beneficial.

Engineering problems contain regularities to varying degrees. For example, attempting to memorize a stream of random numbers is a completely irregular problem, while memorizing a value in a stream of numbers output from a sine function is regular. In contrast to the regular example, numbers in a random stream have no relationships to other numbers in the stream to exploit in the solution. Real world problems are not entirely regular, and effective algorithms must both exploit regularities and handle irregularities to perform well [24]. Indirect encodings have difficulty generating phenotypes with irregular elements, which negatively impacts their performance on irregular problems. [24, 31].

Previous work has shown that EA performance on problems with some regularity and some irregularity can be improved by evolving genomes that combine indirect and direct encodings because the indirect encoding can capture the regularities and the direct encoding can handle the irregularities [24, 32]. Other work has introduced a cell-based indirect encoding called Epigenetic Tracking capable of adapting to both regular and irregular problems by including developmental rules that can target individual cells [33, 34]. While experiments show Epigenetic Tracking can handle irregularities, and in principle can exploit regularity, whether it performs better than a direct encoding on problems with some regularity has not yet been experimentally investigated.

One way of combining indirect and direct encodings is the HybrID algorithm [24, 32], which evolves indirectly encoded genomes until a predefined *switch point* where the genomes are converted to a direct encoding and further evolved to produce irregular features. While HybrID can effectively solve regular and irregular problems, a switch point must be predefined by the user, which is a drawback because the optimal switch point for different problems is not knowable ahead of time and requires substantial computational effort to empirically determine. Also, the final evolved HybrID genomes are directly encoded and thus cannot exploit new regularities that may emerge if the environment changes or the solutions are transferred to a different problem [28, 29]. The strengths and weaknesses of HybrID raise the question of how to best combine indirect and direct encodings to fully exploit the benefits of each encoding, which we investigate by introducing two new HybrID encodings that address specific limitations of the original HybrID method. We report that these new encodings are capable of outperforming the original HybrID for certain classes of problems and shed light on how the strengths of each encoding may be fully employed.

## HyperNEAT and HybrID

All of the algorithms in this section have been previously described in depth, so here we describe them only briefly.

### Compositional pattern producing networks

The natural development process for biological organisms from genetic blueprint to phenotype is an example of indirect encoding on a massive scale. Each cell in an organism has the same genetic code and undergoes a process of cellular differentiation to produce different cell types with specialized functions. Cellular differentiation occurs according to localized chemical gradients that allow the cell to localize itself within the phenotype. The genome can be abstracted as a complex function that takes as input a location and produces as output the description of the phenotype at that location.

*Compositional pattern-producing networks* (CPPNs) are an indirect encoding that abstracts natural developmental processes [35]. A CPPN is a network of nodes that operates much like a neural network, except instead of each node being the same, CPPNs have a small set of different *activation functions* (e.g. sine, Gaussian). Complex geometric functions can be encoded in the CPPN by the composition of these functions, which is defined by the network’s connectivity and weights.

**Fig 1:**
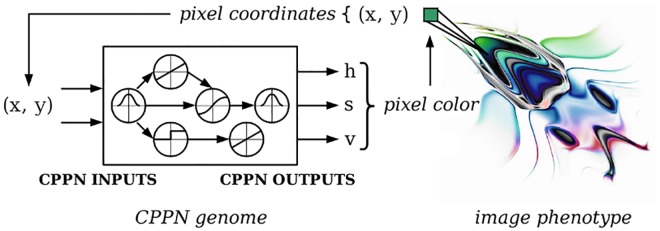
A CPPN genome generates a Picbreeder image phenotype. Adapted from [24]. The *x* and *y* coordinates of a pixel in the phenotype are input to the CPPN, which outputs the hue, saturation, and value components of the pixel color [36]. The process is repeated for all pixels in the phenotype.

To generate a phenotype, the geometric location of each phenotypic element is iteratively input into the CPPN, which outputs the properties of that element. The Picbreeder project [36], which evolves two-dimensional images, is an example of how a CPPN may encode phenotypes (Fig 1). To generate a Picbreeder phenotype from the genome, the *x* and *y* coordinates of a pixel in the image are input to the CPPN, which outputs a color value. The process is repeated for all pixels in the image, producing the complete phenotype. The CPPNs are then evolved to produce interesting new images.

### HyperNEAT

**Fig 2:**
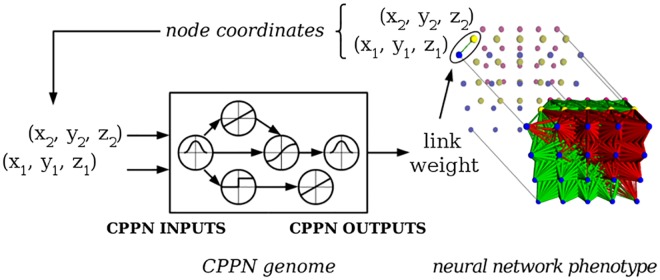
HyperNEAT generates an ANN phenotype. Adapted from [24]. The coordinates of two nodes in the ANN phenotype are input to the CPPN, which outputs the link weight between nodes. The process is repeated for all links allowed in the phenotype. Red links are inhibitory, green links are excitatory, and link weight magnitude is indicated by the thickness of the link.

*Hybercube-based NeuroEvolution of Augmenting Topologies* (HyperNEAT) is a CPPN based indirect encoding for evolving ANNs [20]. The HyperNEAT ANN phenotype is a geometrically arranged substrate of nodes with spatial coordinates. HyperNEAT’s CPPN genome encodes the pattern of connections, or *connectivity*, between ANN nodes (Fig 2). HyperNEAT performs well on many different domains [4, 6, 15–17].

HyperNEAT evolves the CPPN genome via the NEAT algorithm [20, 37]. Genomes are mutated by adding or removing nodes and links, and by modifying link weights. NEAT fine tunes solutions through a process of complexification, in which the genome is initialized with no hidden nodes and increases in complexity as mutations add more links and hidden nodes. NEAT also performs intelligent crossover of genomes during reproduction, and includes speciation to preserve diversity by having individuals only compete with genetically similar individuals [37].

With the CPPN genome, HyperNEAT can create geometric regularities in ANNs that exploit problem regularity [19, 26–28]. Previous work shows that HyperNEAT is sensitive to the geometric representation of the problem, which impacts how effectively HyperNEAT exploits regularity [27]. Indirect encodings typically must increase the complexity of the genome to output more irregular patterns, and as a result, the genome size and the regularity of the phenotype are usually inversely related [24, 38]. For example, the genome only needs to encode a constant value to generate the weights in a completely regular ANN where all link weights are the same. Otherwise, if the ANN is completely irregular, the genome must encode all weight values to generate the phenotype.

### HybrID

**Fig 3:**
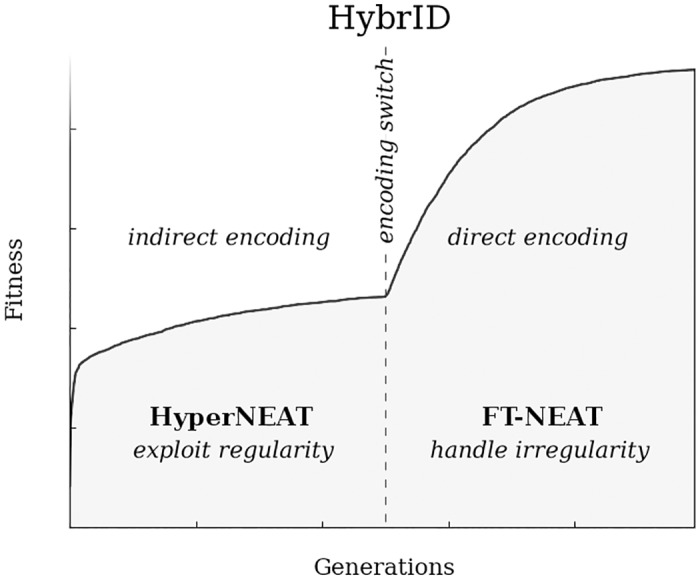
The HybrID algorithm. Individuals are initially indirectly encoded (here, with HyperNEAT) to exploit problem regularities. At the encoding switch point (dashed vertical line), individuals are converted to a direct encoding (here, FT-NEAT) to further improve performance by handling irregularities of the problem. Adapted from [24].

Previous work to improve the performance of indirect encodings on irregular problems resulted in the development of *Hybridized Indirect and Direct encoding* (HybrID), a system that combines HyperNEAT and a direct encoding [32]. We will call the original version of HybrID from [32] *Preset-Switch-HybrID* to distinguish it from methods introduced in this paper. Preset-Switch-HybrID evolves HyperNEAT individuals to generate a regular solution for a fixed number of generations, after which Preset-Switch-HybrID converts the individuals to a directly encoded format, *fixed-topology NEAT* (FT-NEAT), for continued evolution to improve performance on the irregular aspects of the target problem (Fig 3). FT-NEAT directly evolves link weights for fixed-topology ANNs via a modified NEAT algorithm where mutations do not add or remove nodes and links, which is not necessary for the experiments in this paper.

Fitness on problems with irregularity improves much faster after switching to the direct encoding compared to HyperNEAT-only treatments [32] because the direct encoding is better suited for adapting to irregularities by making irregular mutations to the regular phenotype evolved with HyperNEAT [24]. The point at which Preset-Switch-HybrID switches between encodings must be determined by the user, and the optimal switch point can vary depending on how quickly HyperNEAT reaches a fitness plateau after exploiting regularities. Picking a good switch point makes a huge difference in performance, but is not knowable without significant trial-and-error computation. Also, converting the population to a direct encoding after the switch point results in the loss of the compressed, regular genotype produced by HyperNEAT, and consequently the individuals produced lose their ability to exploit regularities on any new problems or environments they are transferred to [28]. The process is largely irreversible because finding an indirect encoding that produces a specific, directly encoded phenotype is itself an extremely difficult search problem with no known algorithm that can solve it reliably.

Following [24], Preset-Switch-HybrID experiments have a switch point of 500 generations. Preset-Switch-HybrID is most effective when the switch point is calibrated to occur soon after the indirect encoding ceases to produce fitness gains on the average problem instance. If the switch point is well chosen, the algorithm will convert the population to the direct encoding after the indirect encoding has fully exploited problem regularity to achieve a good general solution.

## New HybrID variants

### Auto-Switch-HybrID

Our first objective is to test whether a new algorithm, *Auto-Switch-HybrID*, produces better performance across varying levels of problem regularity by automatically computing an effective time to switch between encodings for each individual run of evolution instead of using a fixed switch point for all runs as Preset-Switch-HybrID does. Auto-Switch-HybrID relies on the assumption that a good time to switch from the indirect to direct encoding is when fitness stops improving for a while, which suggests that HyperNEAT has run out of regularity to exploit in the problem and that it is time to let the direct encoding begin making exceptions to the rules HyperNEAT learned. Auto-Switch-HybrID samples the fitness of the current generation’s fittest individual (*champion*) as well as a champion from *delta* generations earlier. If the rate of improvement in fitness falls below a parametrized threshold, shown in Eq 1, the algorithm switches from the indirect encoding to the direct encoding.

$$\begin{array}{c} \frac{{\text{fit}}_{\text{gen}}-{\text{fit}}_{\text{gen}-\text{delta}}}{\text{delta}}<\text{threshold} \end{array}$$ (1)

All of our experiments are performed with an empirically derived threshold of 0.5% improvement vs. delta generations ago, which produces switch points early in runs yet allows enough time for the indirect encoding to effectively exploit problem regularity. The value for fit_gen_ is the mean fitness of the current generation champion and the four preceding generation champions. The value for fit_gen–delta_ is the mean fitness of a generation champion from 100 generations prior and the four subsequent generation champions. A sliding window mean fitness that includes five generation champions minimizes the impact of fluctuations in fitness occurring on a generation-by-generation basis. We empirically determined that setting delta to 100 generations strikes a nice balance between avoiding prematurely switching and delaying a helpful switch.

### Offset-HybrID

Auto-Switch-HybrID solves one of the problems with the original HybrID idea (Preset-Switch-HybrID), which is that it no longer requires manually specifying the switch point. However, it retains another major drawback, which is that there is a one-way, irreversible transfer from an indirect encoding to a direct encoding. That prevents solutions from being able to discover new geometric regularities, either within the original problem or if the problem (or environment) changes.

Thus, a second goal of this paper is to propose and test an additional new version of HybrID that solves both of these problems by evolving indirect and direct encodings simultaneously. *Offset-HybrID* evolves individuals that possess a CPPN genome and a directly encoded genome that are combined to produce the phenotype. The CPPN generates a set of regular values for the link weights, which are then modified by adding corresponding offset values from a directly encoded genome in order to fine tune the network for the specific problem. All offset values in the directly encoded genome are initialized to zero. When a minimum weight magnitude is required to enable a link in the phenotype (whether this is true for each experiment is described below), the CPPN output and offset value are separately compared to the minimum magnitude in order to allow either the CPPN or the direct encoding to activate the link (note: if both are under the threshold the link is not expressed, even if the sum of the two is above the threshold).

Both the indirect CPPN genomic component and the directly encoded offsets component are separately mutated, and separately factor into the NEAT speciation algorithm (with an adjustable parameter that weights the relevance of the indirect vs. direct genomic component in that calculation). Mutation rates for the directly encoded genome are set separately from mutation rates for the indirect encoding. For all experiments, mutations to Offset-HybrID directly-encoded genomes alter on average 7 offsets per genome with a maximum mutation magnitude of 1.17% of the range of the offsets, which preliminary experiments revealed to produce good performance. Because crossing both the directly and indirectly encoded sub-genomes of individuals would produce a higher effective mutation rate, Offset-HybrID performs crossover only on the directly encoded genomes. The indirectly encoded sub-genome is taken whole from one of the parents selected at random and is then probabilistically mutated as usual. The computational costs of Offset-HybrID are roughly the costs of HyperNEAT with the added memory costs of the directly encoded genome, which grows linearly with the number of links in the phenotype, and the cost of mutating each value in the directly encoded genome each generation. These costs are small in comparison to the usual bottleneck in evolutionary algorithms, which is the fitness evaluation, and Offset-HybrID requires no additional computation during this evaluation.

The third goal of this paper is to test whether evolving both encodings simultaneously allows Offset-HybrID to better adapt to new problem domains or changed environments than other HybrID encodings. Because Offset-HybrID retains an indirect encoding throughout evolution, we hypothesize it will be able to continue to exploit regularities when transferred to a new problem, which is especially helpful when the transfer task shares underlying regularities with the original task [28]. We hypothesize that switch-based HybrID methods that convert the population to a direct encoding will not be able to exploit regularity in the new task and should thus not perform as well on new tasks.

After our experiments were completed, but before we had submitted them for publication, a new version of HybrID called R-HybrID was announced [39], which combines HyperNEAT and NEAT by generating some weights in an individual phenotype from a NEAT genome and the rest from a HyperNEAT genome. Phenotype weights may be mutated so they are generated by one genome or the other. The design is similar to Offset-HybrID, but R-HybrID weights are restricted to being either entirely directly encoded, or entirely indirectly encoded, while Offset-HybrID allows the CPPN to create regular weight patterns on top of which exceptions can be made to modify or even completely violate the pattern. Additionally, Offset-HybrID contains both mutations to the CPPN, which can create regular changes to all weights (including those with offsets), and mutations to the direct encoding, which can modify the way each weight deviates from the pattern. We were not able to redo all of our experiments to add this algorithmic variant, but a direct comparison between the two is an interesting area for future research.

## Target weights experiment

### The target weights problem

**Fig 4:**
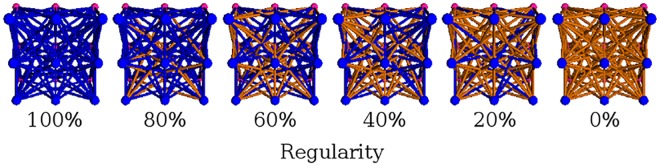
Adjusting the regularity of the target weights experiment. The regularity of the target weights experiment is scaled by adjusting the percentage of repeated link weight values (blue) versus the amount of random link weight values (orange) in the target network. Completely regular instances have 100% repeated values, while completely irregular instances have random values for all link weights. Adapted from [24].

In order to measure the effectiveness of the different encoding methods across multiple levels of regularity, we first test Auto-Switch-HybrID and Offset-HybrID on the *target weights experiment*, first introduced in [31], in which the link weights in an ANN are evolved to match those in a target ANN. We also replicate the results of HyperNEAT and Preset-Switch-HybrID from [24] to set a performance baseline for comparison with the new methods. The target network is a feed-forward network with two layers of nodes (each arranged in a three-by-three grid, Fig 4), and has one layer of (3 × 3) × (3 × 3) = 81 weights, with each weight having a maximum absolute magnitude of 3. No minimum weight magnitude threshold is specified for phenotypes in the target weights experiment. For each individual, the per-link error is calculated by comparing each weight value in the individual with the corresponding weight value in the target network. Error is the sum of the absolute magnitude of the individual weight value differences between networks. Fitness is determined by subtracting an individual’s error from the maximum error, which is the number of target ANN links multiplied by link weight value range.

The regularity of the problem is adjusted by changing the percentage of repeated values versus random values in the target network (Fig 4). It has been previously shown that generative encodings increasingly outperform indirect encodings as the regularity of the Target Weights problem is increased, but that generative encodings fail to handle the irregular exceptions to the regular pattern, hurting performance [24]. We test each level of regularity from 0% regular to 100% regular at intervals of 10% with 40 independent runs of evolution. All experiments have a population of 1,000 individuals and evolved for 2,000 generations.

### Indirect encodings struggle on irregular problems

**Fig 5:**
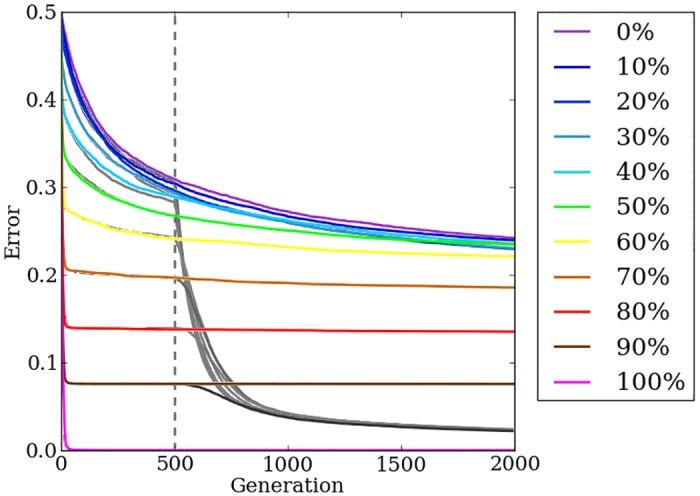
The mean error of HyperNEAT and Preset-Switch-HybrID generation champions over 2,000 generations on the target weights experiment. The line colors indicate the regularity of the problem tested. Preset-Switch-HybrID treatments diverge from HyperNEAT treatments at 500 generations and are gray.

When the target weights pattern is regular and contains many repeated values, HyperNEAT reduces the population’s mean error to minimal amounts within a few generations (Fig 5). Fitness plateaus are notable for all treatments by the 1,000th generation as reductions in the mean error slow dramatically after HyperNEAT can no longer make progress by exploiting regularity and cannot improve the champion solution by any noticeable amount (Fig 5).

Overall, performance on the target weights problem decreases as irregularity increases (Fig 5). On irregular problems, HyperNEAT makes small reductions in error early in evolution and reaches a plateau more slowly than regular problems, but as a result, only regularities greater than or equal to 70% show significant (*p* < 0.0001: All *p* values are generated via the Wilcoxon rank-sum statistical test.) reductions in final error compared to less regular treatments. In this case, and any other in which we perform multiple pairwise comparisons between different treatments to test a hypothesis, such as here comparing each regularity level to all the others, we apply a Bonferroni correction to reduce the risk of false positives. This correction substantially lowers the threshold at which a *p* value is considered significant, and for this reason is often regarded as overly conservative. In cases where a comparison would be considered significant at a *p* < 0.05 threshold, but is no longer at the lower threshold required by the Bonferroni correction, we add NSABC, an acronym for not significant after a Bonferroni correction. As previously demonstrated, HyperNEAT is effective at exploiting problem regularities, but struggles when irregularity is present [24].

### Switching from an indirect encoding to a direct encoding allows HybrID to first exploit problem regularity, then handle problem irregularity

For the 100% regularity condition, the indirect encoding solves the problem in a few generations and well before the switch point. There is thus no difference between HyperNEAT and Preset-Switch-HybrID (Fig 5): error for this treatment is significantly (*p* < 0.0001) lower than all other treatments.

For all problems with some irregularity, immediately after the switch point, mean error for Preset-Switch-HybrID begins to drop: by the final generation, the algorithm has found nearly perfect solutions in all cases (Fig 5). The final error achieved by Preset-Switch-HybrID is nearly identical irrespective of whether the problem is anywhere between 90% regular and 0% regular, demonstrating that the algorithm successfully solves target weights problems without performance greatly degrading on irregular instances (Fig 5). Confirming the results of previous work [32] [24], a comparison of final champion error values from all treatments on problems with irregularity (*n* = 400) shows that Preset-Switch-HybrID (median = 0.0229, ±bootstrap 95% confidence intervals = [0.0215, 0.0232]: All confidence intervals are calculated using the Python scikits.bootstrap module, which by default uses the Bias-Corrected Accelerated Non-Parametric method.) produces significantly (*p* < 0.0001) less error than HyperNEAT (0.2115, [0.1271, 0.2253]) by evolving irregularity in the directly encoded phenotype that is difficult for HyperNEAT to produce. The Preset-Switch-HybrID algorithm demonstrates the benefits of combining direct and indirect encodings by both exploiting regularity when possible and effectively handling irregularity.

### Automation of the encoding switch point allows the most efficient use of both encoding methods

**Fig 6:**
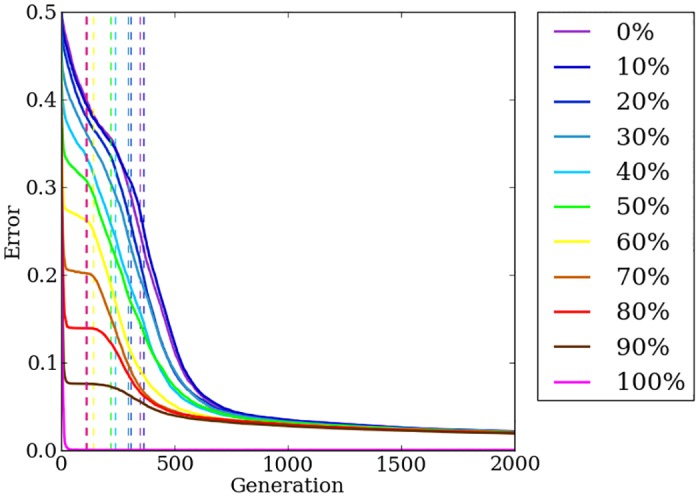
The mean error of Auto-Switch-HybrID generation champions over 2,000 generations on the target weights experiment. The line colors indicate the regularity of the problem tested. Dashed vertical lines indicate the mean switch point per treatment.

Auto-Switch-HybrID automatically calculates the switch point as a function of fitness stagnation and, consequentially, problem regularity (Fig 6). The automatically calculated switch points are earlier than the fixed switch point of Preset-Switch-HybrID (compare Figs 6 to 5), revealing that the indirectly encoded genome quickly exploited problem regularities and then did not further reduce error for a time. Interestingly, the switch point correlates with the regularity of the problem: on regular problems, the available regularity is quickly exploited by the indirect encoding, but on more irregular treatments, the indirect encoding makes incremental reductions in error for a longer period of time, which delays the switch point somewhat (Fig 6).

On 100% regular treatments, the indirect encoding evolves nearly perfect solutions without assistance from the direct encoding and final error values are significantly (*p* < 0.0001) lower than irregular treatments, while final error values for problems with irregularity are similar without performance greatly degrading (Fig 6). The least-regular treatments (0% and 10% regularity) perform worse than more-regular treatments (*p* > 0.0009, NSABC), but overall the performance of Auto-Switch-HybrID does not greatly degrade on irregular target weights instances.

**Fig 7:**
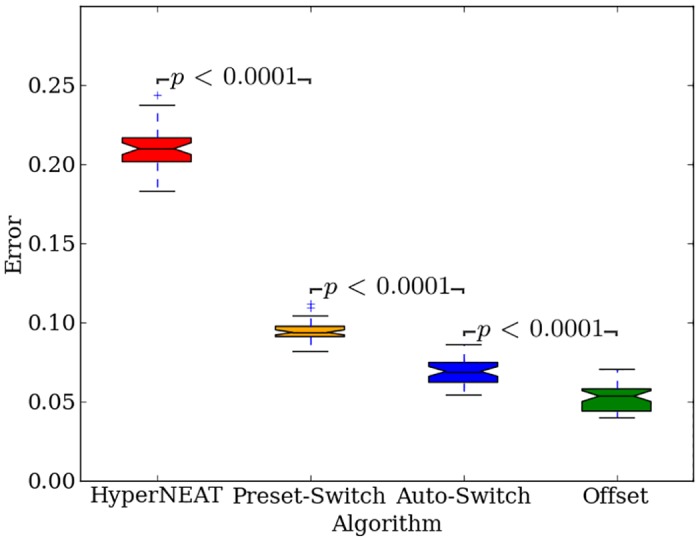
The performance of all methods on the target weights experiment. The mean error of generation champions from the target weights experiment (performance) averaged over all 2,000 generations and pooled across all regularities (*n* = 440) (lower is better). In this and similar plots throughout the paper, each colored box is bounded by the lower and upper quartile values, with a line at the median. The whiskers show the range of the data, while the box notches are proportional to the interquartile range and are calculated using the default matplotlib function.

Pooling across all treatments, the final-champion error values (*n* = 440) for Auto-Switch-HybrID (0.0202, [0.0188, 0.0208], colored lines in Fig 6) are significantly (*p* < 0.0001) less than Preset-Switch-HybrID (0.0229, [0.0219, 0.0232], gray lines in Fig 5) due to the effective switch points calculated by Auto-Switch-HybrID that maximize the benefit of both the indirectly and directly encoded genomes. In addition, the mean error of Auto-Switch-HybrID generation champions averaged over all 2,000 generations and pooled over all regularities (*n* = 440) is significantly (*p* < 0.0001) less than that of Preset-Switch-HybrID, showing that Auto-Switch-HybrID finds better solutions more quickly than Preset-Switch-HybrID (Fig 7). The results show that Preset-Switch-HybrID allows fitness to stagnate with the indirect encoding when switching to the direct encoding is more beneficial.

### Concurrently evolving indirect and direct encodings effectively solves problems across the spectrum of regularity

**Fig 8:**
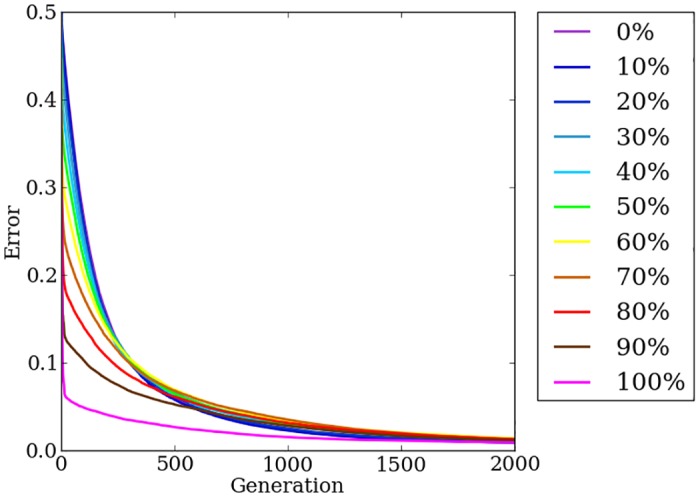
The mean error of Offset-HybrID generation champions over 2,000 generations on the target weights experiment. The line colors indicate the regularity of the problem tested.

Initially on the target weights problem, error levels of Offset-HybrID are similar to HyperNEAT and improve with increasing problem regularity. Roughly before generation 250, mean error is reduced more quickly for irregular problems compared to regular problems (Fig 8). By the 500th generation all regularities except 100% have converged to similar mean error levels (Fig 8). After 2,000 generations, median error for 100% regular treatments is significantly (*p* < 0.0009) less than 30%, 50%, 60%, and 70% regular treatments. Offset-HybrID achieves less error with the 20% regular treatments (*p* > 0.0009, NSABC), and significantly less error with the 10% (*p* < 0.0009), and 0% regular treatments (*p* < 0.0009) compared to more-regular treatments up to 80% regular. It is notable that the least-regular treatments achieve performance comparable with the most-regular treatments. For the target weights problem, Offset-HybrID solves irregularities effectively with the directly encoded genome.

Offset-HybrID significantly (*p* < 0.0001) outperforms other encodings by finding better solutions in fewer generations, measured by the mean error of generation champions averaged over all 2,000 generations and pooled over all regularities (*n* = 440) (Fig 7). The rate at which irregular treatments reduce error suggests that mutations of both the indirect and directly encoded genomes can improve the phenotype’s performance on the problem (Fig 8). Offset-HybrID does not struggle on irregularity like HyperNEAT does and reduces error on irregular treatments much faster as a result (Figs 8 vs. 5). One impact of concurrently evolving direct and indirect encodings is that Offset-HybrID no longer solves 100% regular problems as effectively as HyperNEAT alone (Figs 8 vs. 5). Offset-HybrID’s reduced performance is likely the result of small mutations in the directly encoded genome that bias solutions toward a local optimum early on in evolutionary time.

### The complexity of indirectly encoded genomes increases for irregular problems

**Fig 9:**
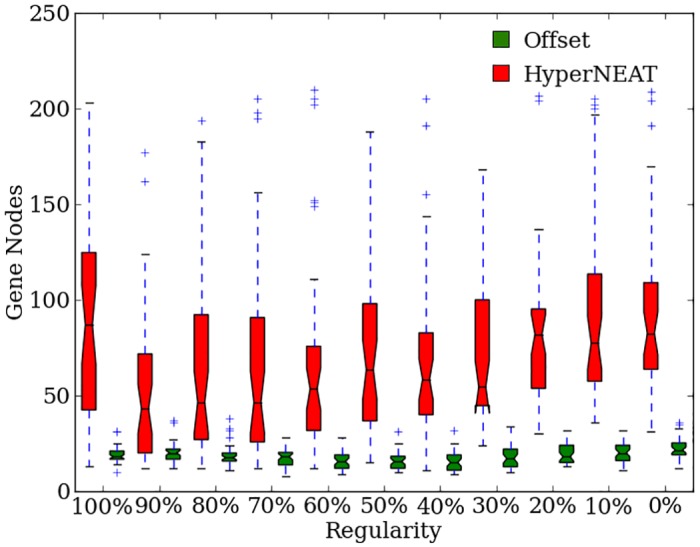
The target weights experiment genome sizes for HyperNEAT and Offset-HybrID. The number of nodes in the CPPN genome of HyperNEAT and Offset-HybrID run champions after 2,000 generations on the target weights experiment.

HyperNEAT improves performance through genome complexification in order to fine-tune solutions and to increase the irregularity of the CPPN output on more irregular problems (Fig 9). In each run of evolution, genomes in the population are initialized with no hidden nodes, which are gradually added to genomes by mutation and crossover. After 2,000 generations, final champion genome sizes tend to increase as problem regularity decreases with the exception of the 100% regular treatments (Fig 9). Genome sizes for 0% and 10% regular treatments are larger than 20% regular treatments (*p* > 0.0009, NSABC). Treatments with 20% to 50% regularity produced larger genomes than more regular treatments (*p* > 0.0009, NSABC). The final genome sizes show HyperNEAT’s tendency to handle irregularity by adding CPPN nodes to increase genome complexity. HyperNEAT displays different behavior on the 100% regular treatments by producing larger genomes than treatments with 40% regularity and higher (*p* > 0.0009, NSABC). Presumably, initial solutions may be found quickly with small genomes on the 100% regular treatments, but the genomes then accumulate nodes that likely do not alter the CPPN output and protect the genome from harmful mutation effects.

### Switching encoding methods allows more efficient evolution of irregular solutions

No simple comparison can be made between the champion *genomes* produced by the switch-based methods and HyperNEAT since the populations of the switch-based methods are directly encoded at the end of runs. However, because regularity relates to the compressibility of a structure [23, 24], we can evaluate the regularity of the final, evolved ANN phenotypes by analyzing compression measured by the *difference* between the size of each phenotype in bits before and after it is algorithmically compressed [21, 24]. We chose the LZW algorithm [40] for this task because it is in the public domain and directly exploits regularity in data to achieve compression.

HyperNEAT phenotypes pooled across all regularities (*n* = 440) are significantly (*p* < 0.0001) more compressible (935 bytes compression, [931, 942]), and thus more regular, than Preset-Switch-HybrID phenotypes (455 bytes compression, [452, 460]). When compared by regularity, HyperNEAT phenotypes are significantly (*p* < 0.0001) more compressible than Preset-Switch-HybrID for all regularities except 100%. The greater irregularity seen in Preset-Switch-HybrID phenotypes confirms that switching to a direct encoding allows for more efficient evolution of irregular phenotypes. HyperNEAT phenotypes are likewise significantly (*p* < 0.0001) more compressible than Auto-Switch-HybrID (459 bytes, [455, 466]), which is mostly similar to Preset-Switch-HybrID, although Preset-Switch-HybrID phenotypes are (*p* > 0.004, NSABC) more compressible for 90% and 100% regular treatments and (*p* > 0.004, NSABC) less compressible for 40% regular treatments. The similarities in compressibility between the switch-based encoding methods demonstrates that Auto-Switch-HybrID is as effective as Preset-Switch-HybrID at evolving irregular phenotypes.

### Presence of a concurrently-evolving direct encoding produces compact, indirectly encoded genomes

Since Offset-HybrID evolves a combined direct and indirect encoding for individuals, the size of the indirectly encoded genome may be compared with HyperNEAT. The indirectly encoded genome for Offset-HybrID champions (24 nodes, [22, 25]) is significantly (*p* < 0.0001) smaller than HyperNEAT (86 nodes, [71, 103]) for all treatments of the target weights problem. Unlike HyperNEAT, the size of the genome does not increase linearly as regularity decreases (Fig 9). Offset-HybrID genome size for 0% regular treatments of the target weights problem is greater than 40% (*p* > 0.0009, NSABC) and 60% regular treatments (*p* > 0.0009, NSABC), and significantly greater than 50% regular treatments (*p* < 0.0009), showing an inverse relationship between genome size and regularity, while above 50% regularity, genome size subtly begins increasing (Fig 9). The sizes of the CPPN genomes evolved by Offset-HybrID suggest that it operates as expected by exploiting regularities with the HyperNEAT genome while the directly encoded genome evolves irregularities (Fig 9). This finding indicates that complexification of the indirectly encoded genome is less important to Offset-HybrID for producing irregular phenotypes.

### Concurrently evolving offsets allow effective evolution of irregularity in the indirectly encoded genome

**Fig 10:**
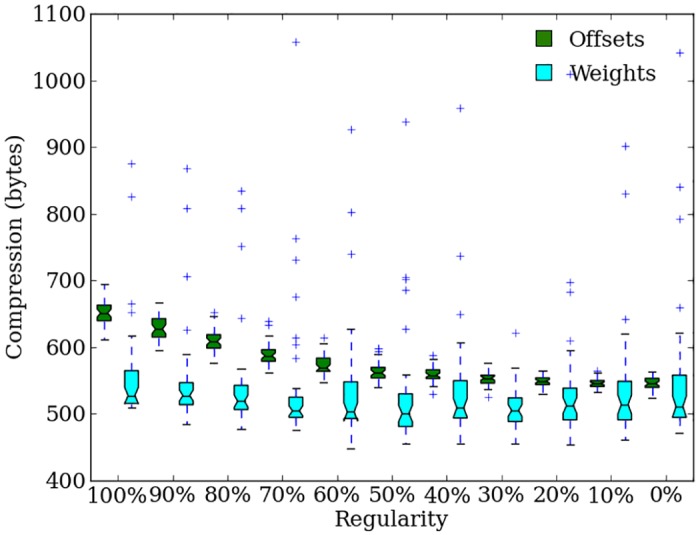
The compressibility of directly encoded offsets and CPPN-generated weights in bytes for Offset-HybrID run champions after 2,000 generations on the target weights experiment higher numbers = more compressible. Offsets are green and CPPN-generated weights are light-blue.

Offset-HybrID phenotypes can also be analyzed with the compression-regularity metric. We separately analyze the compression of the CPPN genome output and the directly encoded offsets, which gives a closer look at how the offsets evolve in relation to the indirect encoding. As problem regularity increases, the offsets become more compressible and regular (Spearman rank correlation, *ρ* = 0.88755, *p* < 0.0001) (Fig 10). The compressibility of the indirectly encoded weights does not vary greatly with regularity, only increasing by small amounts on the most-regular problems (Spearman rank correlation, *ρ* = 0.1589, *p* = 0.0008). The comparison of phenotype compressibility shows Offset-HybrID responding to problem regularity by encoding irregularities with the directly encoded genome.

The compressibility of the directly encoded offsets (564 bytes, [562, 569]) is significantly (*p* < 0.004) greater than the compressibility of the weight values output by the CPPN genome (514 bytes, [510, 517]) (Fig 10). The high compressibility of the offsets is surprising given that the offsets are intended to encode problem irregularities. However, Offset-HybrID’s performance on irregular target weights problems indicates that combining the separately encoded patterns from both genomes produces the irregularity required for a solution (Fig 8).

The compression analysis also reveals that the CPPN-encoded weights of Offset-HybrID phenotypes (*n* = 440) (514 bytes, [510, 517]) are significantly less compressible (*p* < 0.0001) than weights evolved with HyperNEAT alone (935 bytes, [931, 942]). As noted previously, HyperNEAT consistently produced larger genomes than Offset-HybrID on the target weights problem (Fig 9). The two results show that some of the genome complexification by HyperNEAT has little effect on phenotype compressibility, while concurrently evolving indirect and direct encodings produces more-irregular phenotypes with smaller genomes. In initial generations, randomly evolved offsets may disrupt the regular pattern produced by the CPPN, causing evolution to alter the CPPN to compliment the offset and produce a fit solution. The presence of the directly encoded genome allows for more phenotypic irregularity without needing to add more genes into the CPPN genome.

## Bit mirroring experiment

### Description

To better understand how well the different encodings handle irregularity, it is necessary to test them on problems more difficult than the target weights experiment, which has no epistasis (interactions between parameters), and thus no local optima. In the *bit mirroring experiment* [31], a feed-forward ANN is evolved to mirror bits, input to each node in the input layer, to a specific corresponding node in the output layer. This problem is more complicated than targets weights, as will be described below.

The target network consists of two layers, each with 49 nodes geometrically arranged in seven rows and seven columns. Links in the target network can be constrained to columns or rows within the two-dimensional layout of the layer. Phenotype links are enabled if the absolute magnitude of the CPPN weight output is greater than 0.2, and links weights are normalized to have an absolute magnitude within the range 0 to 3. Just before publication, we noticed a small error in the code that allowed Offset-HybrID treatments to have weights outside of this prescribed range. We do not believe this would qualitatively change our conclusions.

To evaluate fitness, an individual network is given a random set of bits as input that propagate through the network and the output is checked for correctness. The individual’s fitness value is incremented for each node in the output layer that mirrors the correct input bit according to the target solution. Because the network input is random, each network is tested ten times with different inputs and fitness is summed across all tests in order to ensure that fit networks are truly solving the problem rather than producing a solution for one random input by chance. In contrast with the target weights experiment, which evolves ANNs toward a goal by comparing network topologies, the bit mirroring experiment evolves ANNs toward a goal by comparing network input-output mappings. Many differently wired networks may produce similar output, which is another reason that the fitness space of the bit mirroring problem is more complex than target weights. Correctly mirroring a bit requires that the link between the input and target nodes be turned on while other connections to the output node are turned off. The output value for a node at the endpoint of a correctly wired link may be incorrect due to the presence of other links, which is an example of *epistasis* and makes the problem more difficult to solve.

**Fig 11:**
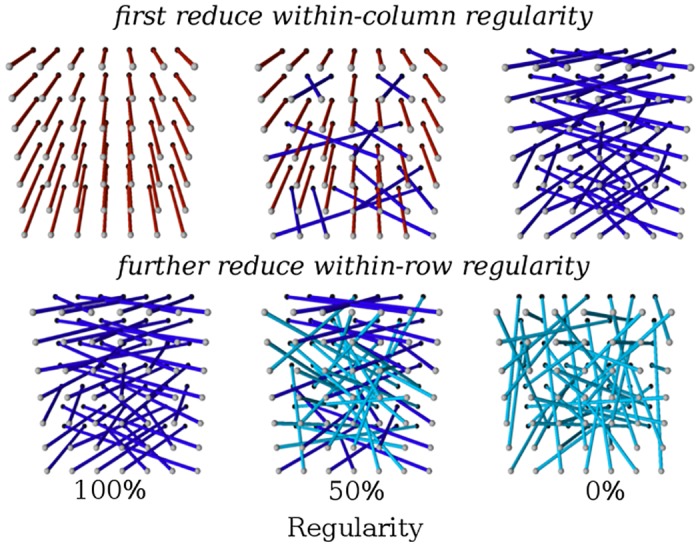
Adjusting the regularity of the bit mirroring experiment. Completely regular target networks for the bit mirroring experiment directly mirror bits from input layer nodes to corresponding nodes in the same row and column of the output layer. Regularity is decreased by first releasing the constraint that nodes connect within the same column, then by releasing the constraint that nodes connect within the same row. Red links are row-and-column constrained, blue links are row constrained, and light blue links are unconstrained. Adapted from [24].

The most-regular networks have each node in the input layer directly wired to the corresponding node in the same row and column of the output layer. Regularity can be reduced by first relaxing the constraint that input nodes are wired to the same column in the output layer, then by relaxing the within-row constraint so that the most-irregular networks require that bits be mirrored to random nodes in the output layer (i.e. 0% column-constrained and 0% row-constrained) (Fig 11). We perform the bit mirroring experiment at regularities ranging from 0% to 100% row-constrained and 0% to 100% row-and-column-constrained at intervals of 10%. Each level of regularity is tested with 40 treatments of evolution. All encodings were evaluated with a population of 1,000 individuals and evolved for 2,000 generations. The parameters for the experiment have been changed slightly from the parameters used in [24] to limit the growth of HyperNEAT’s genome size by reducing the chance that mutations add gene nodes. Like the target weights experiment, we replicate the results of HyperNEAT and Preset-Switch-HybrID from [24] for comparison with the new methods.

### Indirect encodings consistently struggle on irregularity

**Fig 12:**
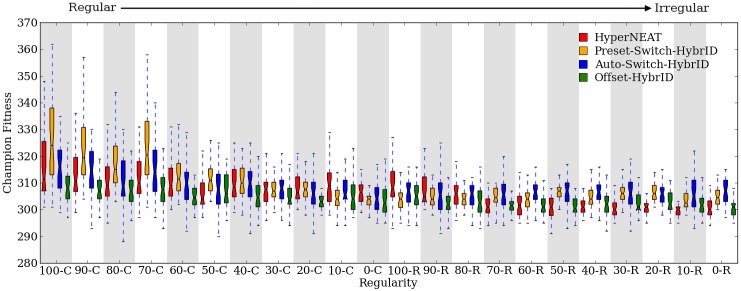
Final fitness per regularity for all methods on the bit mirroring experiment. The fitness of run champions after 2,000 generations for each regularity of the bit mirroring experiment.

HyperNEAT’s performance on the bit mirroring experiment is impacted by problem regularity in the same fashion as the target weights experiment. On regular problems, HyperNEAT is able to solve major regularities early resulting in large gains in median fitness followed by steadily improving fitness through the remaining generations. 100% regular treatments achieved higher fitness than other regularities (*p* > 0.0002, NSABC) except 90% and 70% column-regular treatments (Fig 12). As problem irregularity increases, initial fitness gains are reduced as HyperNEAT is able to exploit problem regularity to a lesser degree (Fig 12).

On problems with irregular elements, improvements in fitness become infrequent after the 100th generation when HyperNEAT has exploited most problem regularities. The fitness of run champions on irregular problems less than 60% row-constrained is not significantly (*p* > 0.05) different at the 100th generation (294, [293.99, 295]) compared to the 2,000th and final generation (294, [293.99, 294.01]), demonstrating that few fitness gains occur after initial generations in which HyperNEAT exploits underlying problem regularities. The plateau effect on fitness becomes more pronounced for irregular problems compared to regular problems, resulting in decreasing final median fitness as problem irregularity increases (Fig 12).

### Switching encoding methods benefits fitness

**Fig 13:**
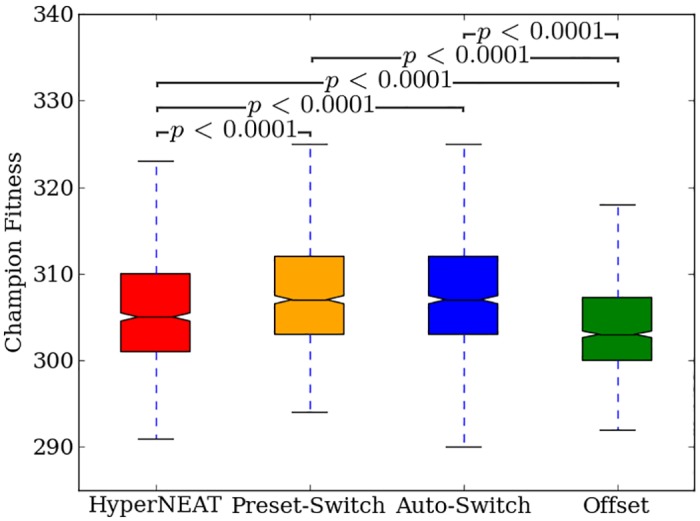
Final fitness for all methods on the bit mirroring experiment. The fitness of run champions after 2,000 generations pooled across all regularities of the bit mirroring problem.

Comparing the final fitness of all run champions pooled across all regularities (*n* = 880) shows that Preset-Switch-HybrID (304.97, [303.94, 304.97]) significantly (*p* < 0.05) outperforms HyperNEAT (302.98, [301.99, 303.04]) on the bit mirroring experiment (Fig 13).

As noted previously, the bit mirroring problem exhibits epistasis because the fitness benefit of correctly wired links depends on other links. To solve the problem, each output node must be wired to its corresponding input node and no other. Evolution must find the pattern of necessary input-to-output connections, which is dependent on problem regularity, and must also remove unnecessary links to the output nodes, which is a fundamentally regular change repeated for each node, but is also dependent on the problem regularity. Due to the increased problem difficulty, the performance of Preset-Switch-HybrID for bit mirroring (Fig 12) is much more dependent on problem regularity than for the target weights problem (Fig 5).

### Automated encoding switching performs as well as preset switching

Using the same 0.5% fitness-improvement switch threshold as the target weights experiment, Auto-Switch-HybrID tends to choose switch points in early generations for the bit mirroring experiment (131 gens, [129, 132]). The switch points are roughly clustered together soon after 100 generations, which is the earliest that Auto-Switch-HybrID may switch. The indirect encoding’s performance on most problems quickly reaches a plateau, triggering the switch to the direct encoding. Early switch points appear to be most beneficial for irregular problems, which the direct encoding is better suited to solving (Fig 12). On the most-regular problems, the early switch points can prevent the indirect encoding from solving regularities that would allow for fitness gains if given more time before the encoding switch, while Preset-Switch-HybrID avoids this issue (Fig 12).

A comparison of run champion final fitness values pooled across all regularities (n = 880) shows that Auto-Switch-HybrID (305.01, [304.96, 306.08]) significantly (*p* < 0.05) outperforms HyperNEAT (302.98, [301.99, 303.04]), and is not significantly different (*p* > 0.05) than Preset-Switch-HybrID (304.97, [303.94, 304.97]) (Fig 13).

The final fitness values for 100% regular treatments were (*p* > 0.0002, NSABC) greater than other regularities, except 90% and 70% column-regular treatments (Fig 12). Auto-Switch-HybrID performs worst on problems of intermediate regularity and improves performance slightly as problems become more or less regular. On problems with high regularity, this is likely due to the indirect encoding finding the regular solution quickly resulting in large fitness gains early. On irregular problems, Auto-Switch-HybrID switches encodings early in the run, allowing more time for the slow fine-tuning process of evolving the direct encoding.

### Concurrently evolving encodings is not an effective strategy for bit mirroring

Offset-HybrID is not as effective on the bit mirroring experiment as the target weights experiment (Fig 13). Offset-HybrID performs most competitively on irregular problems and outperforms (*p* > 0.002, NSABC) HyperNEAT on 10%, 20%, and 30% row-regular treatments (Fig 12). Switch-based HybrID methods outperform Offset-HybrID on all regularities (Fig 12): The final fitnesses of Offset-HybrID run champions pooled across regularities (n = 880) (301, [300.99, 301.01]) are significantly (*p* < 0.0001) less than HyperNEAT (302.98, [301.99, 303.04]), Preset-Switch-HybrID (304.97, [303.94, 304.97]), and Auto-Switch-HybrID (305.01, [304.96, 306.08]).

Offset-HybrID must separately mutate both genomes to account for epistasis in the bit mirroring problem, which makes the problem difficult. Individual ANN phenotypes are produced by summing the patterns encoded in the offsets and the CPPN genome and evaluated according to the ANN output. The patterns from each genome must compliment each other to produce fitter individuals. The bit mirroring fitness landscape is much harder for the concurrently-evolving genomes to navigate than the fitness landscape for the target weights experiment, where weights are directly evaluated for fitness without epistatic effects.

Comparing HyperNEAT genome sizes across regularities shows a subtle and significant trend toward larger genomes as problem regularity decreases (Spearman rank correlation, *ρ* = 0.32, *p* < 0.0001), which is consistent with the results of the target weights experiment and further demonstrates how HyperNEAT attempts to deal with irregularity through increasing genome complexity. Small genomes are useful for producing repeated weight patterns that aid in solving regular bit mirroring problems. Larger genomes are needed to avoid reuse of genomic information, which produces regular phenotypes that are unable to solve irregular problems.

Final champion indirectly encoded genome sizes of Offset-HybrID (24 nodes, [23, 25]) are significantly (*p* < 0.0001) smaller than HyperNEAT (45 nodes, [44, 46]) for all regularities. The presence of a concurrently-evolving, directly encoded genome limits growth of the CPPN genome. The fact that Offset-HybrID produces small CPPN genomes for the bit mirroring problem while failing to outperform HyperNEAT suggests that the direct encoding appears to be unable to effectively encode bit mirroring problem irregularities.

## Quadruped robot experiment

### Description

While the bit mirroring and target weights experiments demonstrate how the encoding methods respond to regularity in the problem structure, they are not representative of the types of real-world problems to which EAs are applied, thus our next objective is to test the encodings on a problem of varying regularity with real-world applications. Following [15, 24], in the *quadruped robot experiment*, an ANN controller for a quadruped robot with twelve joints and nineteen sensors is evolved to walk without falling on a simple flat plane simulated in the ODE physics engine [www.ode.org].

**Fig 14:**
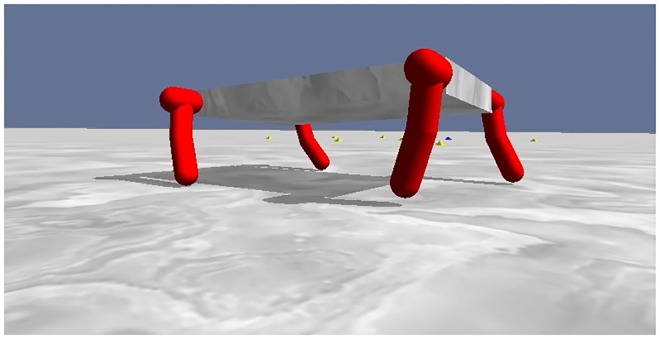
The quadruped robot. The physical design of the quadruped robot.

The robot has a basic design allowing for quadruped locomotion. The torso of the robot is a wide flat box measuring 0.15 (arbitrary) units wide, 0.3 units long, and 0.05 units tall, parallel to the ground plane (Fig 14). Each leg is composed of three cylinders, measuring 0.075 units long with a radius of 0.02 units, and three joints. The first cylinder acts as the leg’s coxa and protrudes slightly from the main body of the robot, while the second cylinder functions as the femur, and the third cylinder as the tibia. The first joint is between the coxa and femur, allowing forward and backward leg rotation constrained to a maximum of 180 degrees. The second joint is also between the coxa and femur and allows rotation of the leg outward from the body. The first two joints work in concert to form a universal hip joint. The third joint is located at the knee and allows forward and backward rotation of the tibia. Touch sensors are located at the end of each tibia, allowing for detection of whether a leg is contacting the ground.

**Fig 15:**
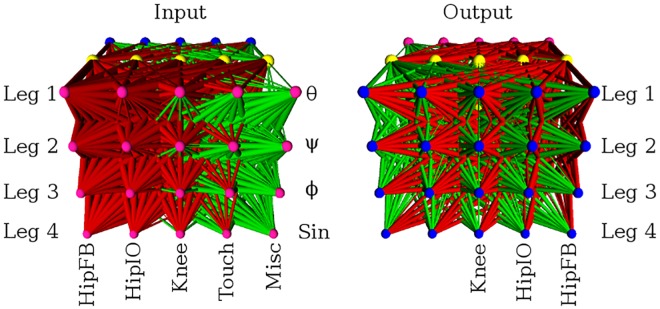
The configuration of the ANN controller evolved for the quadruped robot experiment. *θ*, *ψ*, and *ϕ* are the pitch, yaw, and roll angles of the main body respectively. Red links are inhibitory, green links are excitatory, and link weight magnitude is indicated by the thickness of the link. Adapted from [24].

The ANN controller is a three-layer feed-forward network with input, hidden, and output layers each consisting of twenty nodes arranged in rows and columns (Fig 15). Links may only form between adjacent layers and recurrent links are not allowed. Link weights are normalized to have an absolute magnitude within the range 0 to 3. In this quadruped experiment, there is no minimum weight magnitude threshold under which weights are not expressed. Leg joint angles, leg contact feedback, and the torso’s orientation with respect to gravity are input to the controller. A sine wave is also given as input to facilitate the production of periodic movements. The output of the network is a set of joint angles sent as motor commands to the simulated robot. Due to the layout of the network, two output columns are left unused since only twelve outputs are necessary to control the robot. Since HyperNEAT has been shown to be sensitive to the geometric representation of this problem [27], the inputs and outputs are arranged in the same manner as [15].

Following [15, 24], the fitness of an individual is given by Eq 2:

$$\begin{array}{c} 2^{\left(x^{2}+y^{2}\right)} \end{array}$$ (2)

where *x* and *y* are the distance in ODE units traveled in the x and y dimensions along the ground plane from the starting location. The fitness metric encourages evolution of walking patterns that avoid falling and maximize movement speed through the environment, and it is exponential to emphasize small gains in distance traveled.

Following [15, 24], the regularity of the problem is adjusted by adding some amount of uniformly distributed random error (aka noise) to the joint-angle motor commands received by the robot’s limbs so that the true joint angle in the physics simulation will be offset from the angle output by the ANN, resulting in irregular movement. The joint error ranges from -2.5 to 2.5 radians for all quadruped robot experiments. The simulated irregularities are an abstraction of real-life problems that physical robots encounter, such as weak servos or faulty power supplies. The evolving networks must perceive and compensate for these irregularities or the robot will fall and receive a low fitness evaluation. As in [15] and [24], we perform the quadruped experiment at five levels of regularity using zero, one, two, eight, and twelve noisy joints that are randomly selected at the beginning of the evaluation. Each level of regularity is tested with 40 treatments of evolution. All encodings were evolved for 1,000 generations using a population size of 150 individuals. Results for HyperNEAT and Preset-Switch-HybrID are replicated from [24] for comparison with the new methods.

### Indirect encoding effectively exploits quadruped regularities, but struggles on irregular problems

**Fig 16:**
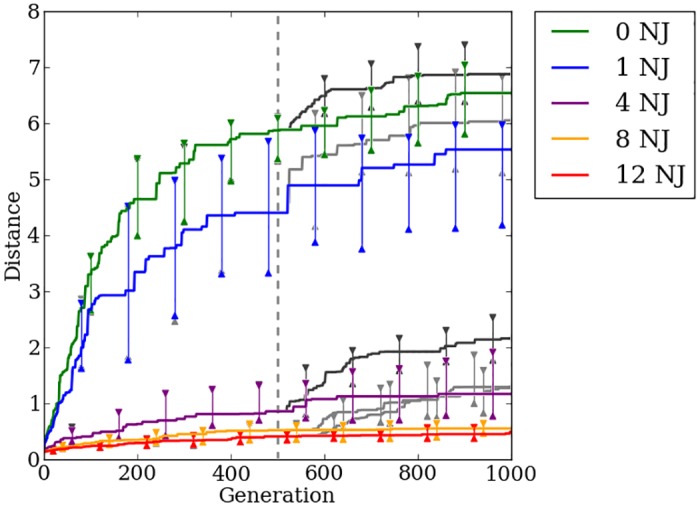
The median fitness for HyperNEAT and Preset-Switch-HybrID generation champions over 1,000 generations on the quadruped robot experiment. The line colors indicate the number of noisy joints on the robot. Preset-Switch-HybrID treatments diverge from HyperNEAT treatments at 500 generations and are gray. 95% bootstrapped confidence intervals of the median are shown every 100 generations.

HyperNEAT is able to exploit the regularities present within the problem to a great degree. On regular problems HyperNEAT can quickly find a stable gait allowing the robot to walk greater distances without falling (Fig 16). As was shown in [15, 24], increasing the number of faulty joints present on the robot results in decreased performance. By the 500th generation on the quadruped robot problem, the rate of improvement in HyperNEAT’s median fitness slows greatly (Fig 16). HyperNEAT continues to make progress after 500 generations, but the rate of improvement is small, which validates that the switch point of 500 generations, as used in [24], is a reasonable point for Preset-Switch-HybrID to switch encodings. Median distance traveled for generation champions on the most-irregular problems with four, eight, and twelve noisy joints improves little over the first 500 generations compared to problems with zero and one noisy joint (Fig 16). Confirming the previous work in [24], we conclude that HyperNEAT is unable to effectively evolve irregularities necessary to produce stable gaits on irregular problems.

### Switching encoding methods handles irregularity to produce fitter walking gaits

As reported in [24], after Preset-Switch-HybrID switches encoding methods, median fitness begins improving at a rate greater than HyperNEAT for all regularities (Fig 16). The gains in median fitness continue as the direct encoding fine-tunes the solution for the final 500 generations. Comparing the final champion fitness values pooled across all regularities (*n* = 200) reveals that Preset-Switch-HybrID (2.58, [2.22, 3.11]) significantly (*p* < 0.05) outperforms HyperNEAT (1.43, [0.91, 2.11]). The final champion fitness values for Preset-Switch-HybrID on the quadruped problem are greater (*p* > 0.01, NSABC) than HyperNEAT for treatments with four, eight, and twelve noisy joints (Fig 16).

Like the bit mirroring experiment (Fig 12), the median fitness values across regularities for Preset-Switch-HybrID encodings do not converge to similar levels by the final generation (Fig 16), in contrast to the target weights experiment (Fig 5). However, the benefit of the directly encoded genome on the quadruped robot experiment is apparent in the rapid increase in median fitness values post-switch for Preset-Switch-HybrID (Fig 16).

**Fig 17:**
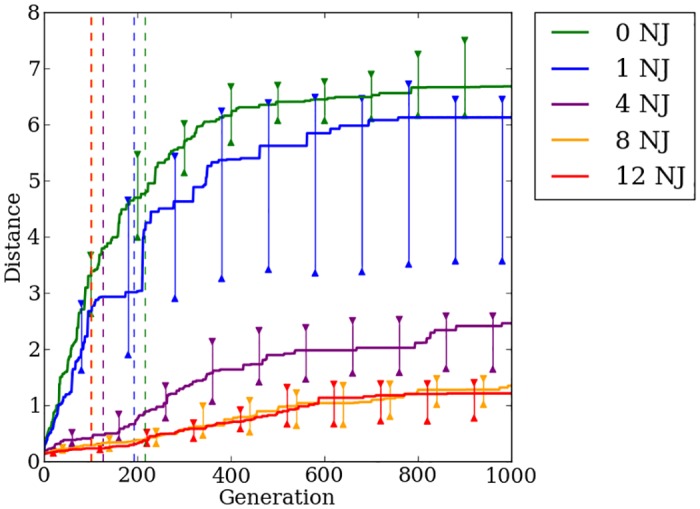
The median distance traveled by Auto-Switch-HybrID generation champions over 1,000 generations on the quadruped robot experiment. The line colors indicate the number of noisy joints on the robot. The median switch points per treatment are indicated by dashed lines. 95% bootstrapped confidence intervals of the median are shown every 100 generations.

Auto-Switch-HybrID switches encodings early on for each regularity (148 gens, [133, 162]) (Fig 17). As with target weights, median switch points for irregular treatments are earlier than regular treatments (Fig 17). The rate of improvement in distance traveled slows quickly for irregular treatments, resulting in early fitness plateaus that trigger the switch. On more-regular treatments, the indirect encoding is able to make steady improvements to performance for a longer period of time thereby delaying the switch point.

**Fig 18:**
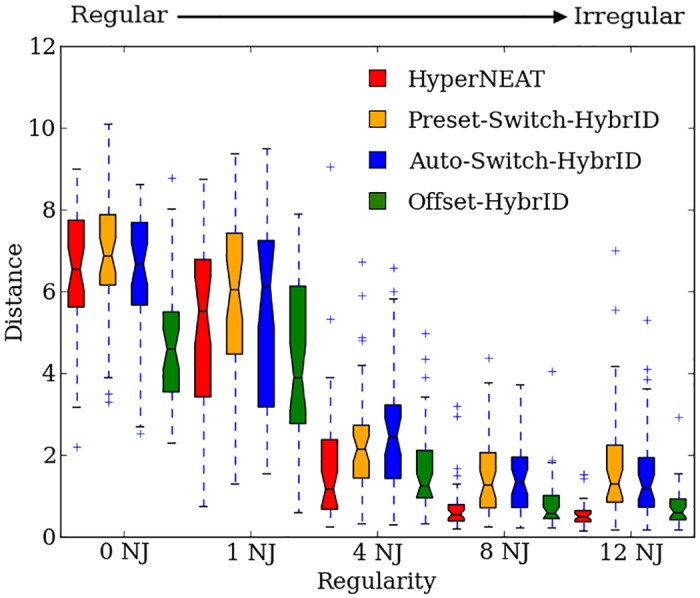
Final fitness per regularity for all methods on the quadruped robot experiment. The distance traveled by run champions after 1,000 generations for each regularity (number of noisy joints) of the quadruped robot experiment.

The overall walking distance achieved by the robot decreases as the problem regularity decreases, but all treatments see marked increases in performance after the encoding switch (Fig 17). Final champion fitness values pooled over all regularities (*n* = 200) for Auto-Switch-HybrID (2.55, [2.25, 3.09]) are not significantly (*p* > 0.05) different from Preset-Switch-HybrID (2.58, [2.22, 3.11]), but are significantly (*p* < 0.05) greater than HyperNEAT (1.43, [0.91, 2.11]). Auto-Switch-HybrID and Preset-Switch-HybrID increasingly outperform the other encodings as regularity decreases (Fig 18). For all experiments comparing switch-based HybrID encodings, Auto-Switch-HybrID matches the performance of Preset-Switch-HybrID while avoiding the need to pre-specify encoding switch points. By increasing the automation of the evolution of irregular solutions, Auto-Switch-HybrID appears to be a more useful encoding than Preset-Switch-HybrID.

### Concurrently evolving encodings provides no fitness advantage over indirect encoding

**Fig 19:**
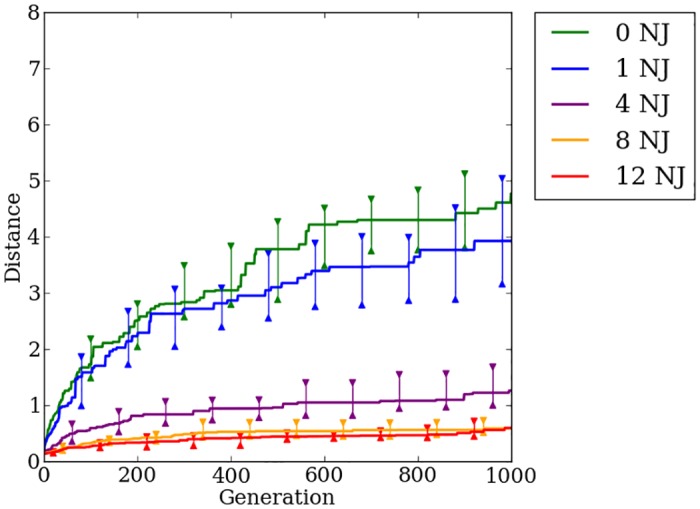
The median distance traveled by Offset-HybrID generation champions over 1,000 generations on the quadruped robot experiment. The line colors indicate the number of noisy joints on the robot. 95% bootstrapped confidence intervals of the median are shown every 100 generations.

Offset-HybrID performs best on regular problems with performance decreasing as the problem irregularity increases (Fig 19). On the most-regular problems, the greatest fitness gains are made early in the evolutionary timescale. On the most-irregular problems, fitness slowly increases throughout the entire 1,000 generation period. Final champion fitness values for Offset-HybrID pooled across all regularities (*n* = 200) (1.48, [1.07, 2.11]) were not significantly different (*p* > 0.05) from HyperNEAT (1.43, [0.91, 2.11]), but were significantly (*p* < 0.05) worse than Auto-Switch-HybrID (2.55, [2.25, 3.09]) and Preset-Switch-HybrID (2.58, [2.22, 3.11]) (Fig 18). Offset-HybrID is generally not able to effectively concurrently evolve the directly encoded genome to perform well on the quadruped robot experiment.

### Regular weight patterns produce regular solutions

**Fig 20:**
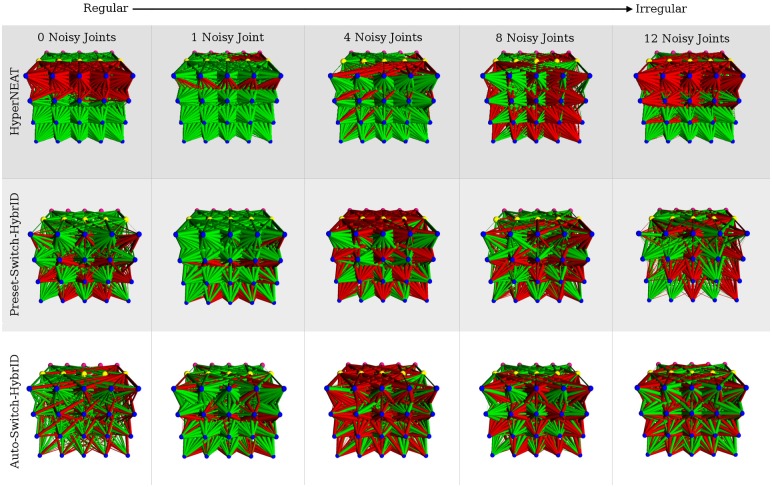
The fittest HyperNEAT, Auto-Switch-HybrID, and Preset-Switch-HybrID ANNs from each regularity of the quadruped robot experiment viewed from the output layer. Red links are inhibitory, green links are excitatory, and link weight magnitude is indicated by the thickness of the link.

3D visualizations of the fittest ANNs produced by HyperNEAT display evolved structural regularity in the form of repetition, symmetry, and interesting patterns in the network link weights (Fig 20). The networks are not visually distinguishable as more or less regular as the regularity of the problems decrease. The 3D renderings of the top performing networks produced by Preset-Switch-HybrID and Auto-Switch-HybrID contain more complex patterns compared to the HyperNEAT networks evolved for the quadruped robot experiment, which displayed simpler regularities and symmetries (Fig 20). The images demonstrate how the encoding-switching methods directly manipulate the phenotype to achieve irregularity and to optimize performance on the specific problem. Evolving the directly encoded networks results in mutations to the network topology that are not coordinated and regular. As with the HyperNEAT networks, it is difficult to visually distinguish if the regularity of the networks increases as problem regularity increases.

### Offset weight patterns are irregular with low weight magnitude

**Fig 21:**
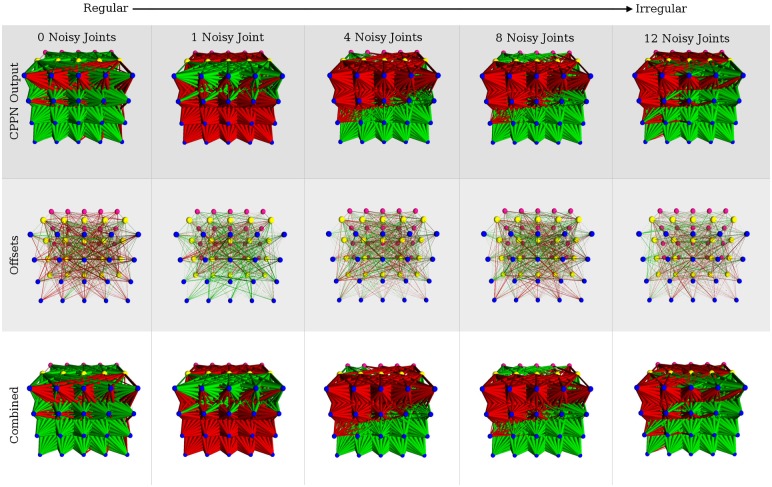
The fittest Offset-HybrID ANNs from each regularity of the quadruped robot experiment viewed from the output layer. Red links are inhibitory, green links are excitatory, and link weight magnitude is indicated by the thickness of the link. A small and significant negative correlation was found between offset magnitude and problem regularity (Spearman rank correlation, *ρ* = −0.293, *p* < 0.0001).

The evolved Offset-HybrID networks exhibit some notable features resulting from the concurrent evolution of multiple encodings (Fig 21). The indirectly encoded phenotype is regular, exhibiting many repeating patterns of weights. 3D renderings of the ANN phenotypes clearly show Offset-HybrID exploiting regularities with the indirect encoding while producing irregular offsets (Fig 21). The offset weight patterns visually appear irregular with few repeated connection patterns. The summed weight magnitude of directly encoded offsets (90.61, [88.54, 92.99]) is small compared to the summed magnitude of indirectly encoded weights (1377.17, [1329.81, 1447.64]) for each individual. The combined phenotypes do not display many noticeable changes in connectivity, such as connections changing from excitatory to inhibitory or vice versa (Fig 21).

## Quadruped robot transferability experiment

### Description

Part of the challenge of evolving ANNs for robots operating in the physical world is handling a dynamic environment where changes may fundamentally alter the steps necessary to accomplish an action, or may result in an entirely new set of goals. Individuals transferred to a new environment are most effective if they preserve existing knowledge and skills and adapt them to the current task, which is known as *transfer learning* [28, 41]. Previous work with HyperNEAT showed that indirect encodings were more effective at transferring knowledge across domains and adapting to new tasks than direct encodings because indirect encodings are capable of encoding relationships between elements of a problem, which allows for greater generalization on problems and better evolvability [28].

Because Offset-HybrID evolves both indirectly and directly encoded genomes, it should be able to transfer to new tasks easier than switching-HybrID methods that preserve only a directly encoded genome after evolution. To demonstrate how encodings transfer to new tasks, a second test of the quadruped robot experiment was performed on previously-evolved populations from Auto-Switch-HybrID and Offset-HybrID. Physical parameters for the robot and environment are identical to the previous quadruped experiments. All populations are evolved in a new quadruped robot experiment for 3,000 generations across the spectrum of regularity, and each level of regularity is tested with 40 treatments of evolution that continue from where the prior quadruped experiment ended. The populations evolve for three times as long as the previous quadruped robot experiments in order to highlight the differences in how Offset-HybrID and Auto-Switch-HybrID (post-encoding-switch) behave over time.

In the new experiment, a regular change is made to the amount of error present in the robot’s joint angles from the previous evolutionary environment by adding a random uniform amount of error to all joints. The added error acts as a new problem that controllers must evolve to solve, and it is intended to model challenges faced by physical robots that affect all joins simultaneously, such as failing power supplies, weak batteries, or altered environmental conditions. Since the change made to joint angle error is uniform and regular, the relationships between joints from the previous problem are preserved. An indirect encoding should be able to quickly adjust to the regular change, unlike a direct encoding. Therefore we hypothesize that Offset-HybrID will outperform Auto-Switch-HybrID (post-encoding-switch) when transferred to the new problem.

Offset-HybrID is capable of retaining irregularities solved in the pre-transfer experiment, while evolving the indirectly encoded genome to solve the new, regular change in the transfer environment. We would also expect HyperNEAT-alone treatments to handle the regular change in the new, transfer environment effectively, however the irregularities from the original problem, which HyperNEAT struggles to solve, remain in the transfer experiment, which could harm HyperNEAT’s performance. Since the point of this transfer experiment is to only test whether maintaining an indirect encoding benefits transferability rather than find the best performing encoding, and due to restricted computational resources, HyperNEAT-alone is not tested on the transfer experiment.

The starting point for evolution on the pre-transfer problem is notably different from the transfer problem. In the most-regular treatments of the pre-transfer problem, the robot has no joints with error, and the problem of controlling the robot is thus simpler. Even in the most-regular treatments of the transfer problem, all joints contain some amount of error that must be corrected in order for the robot to contact the ground properly, which results in a problem that is potentially harder than the pre-transfer problem.

### Switch-based encodings have low transferability

**Fig 22:**
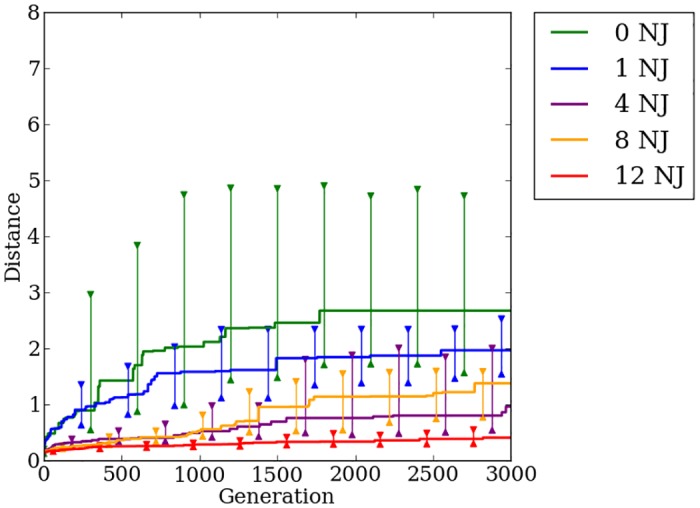
The median distance traveled by Auto-Switch-HybrID generation champions over 3,000 generations on the quadruped robot transfer experiment. The line colors indicate the number of noisy joints on the robot. 95% bootstrapped confidence intervals of the median are shown every 300 generations.

On the first generation of the transfer experiment, Auto-Switch-HybrID gaits evolved for the pre-transfer problem do not perform well with the added joint error, and median distance traveled by the robot is close to zero (Fig 22). The uniform joint error that is added for the new problem disrupts evolved walking patterns, and Auto-Switch-HybrID individuals must undergo further evolution to walk without falling. For the most-irregular treatments, the champion median distance traveled does not improve much over 3,000 generations. The distance traveled improves with regularity, which could mean Auto-Switch-HybrID retains some of the regular patterns evolved by the indirect encoding prior to the transfer problem (Fig 22). The distance traveled after 3,000 generations (*n* = 200) (1.46, [1, 1.66]) is still significantly (*p* < 0.0001) below what Auto-Switch-HybrID was capable of achieving during the pre-transfer problem in 1,000 generations (2.55, [2.25, 3.1]) (Figs 22 vs 17).

### Concurrently evolving encodings retains the advantages of indirect encoding for transfer problems

**Fig 23:**
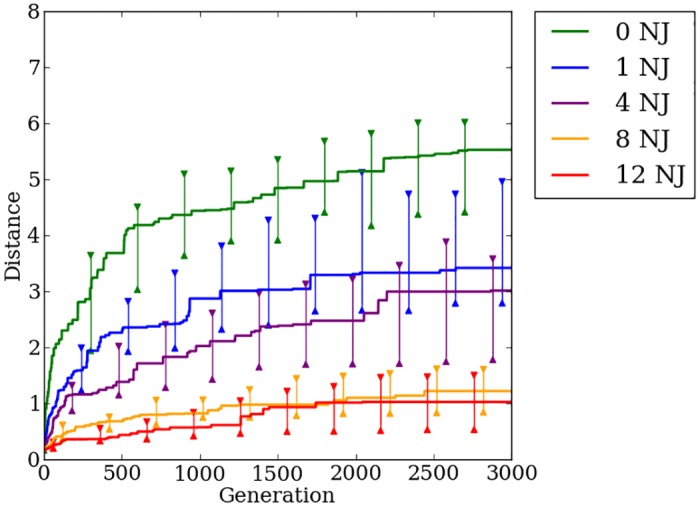
The median distance traveled by Offset-HybrID generation champions over 3,000 generations on the quadruped robot transfer experiment. The line colors indicate the number of noisy joints on the robot. 95% bootstrapped confidence intervals of the median are shown every 300 generations.

Similar to the Auto-Switch-HybrID results, Offset-HybrID gaits evolved for the pre-transfer problem perform poorly on the first generation of the transfer problem (Fig 23). However, Offset-HybrID’s performance improves more quickly on regular problems (Fig 23, 0, 1, and 4 noisy joints). This substantial improvement in distance traveled over time demonstrates the benefit of retaining the indirectly encoded genome. For the irregular treatments with twelve and eight noisy joints, there is less benefit of retaining the indirect encoding.

**Fig 24:**
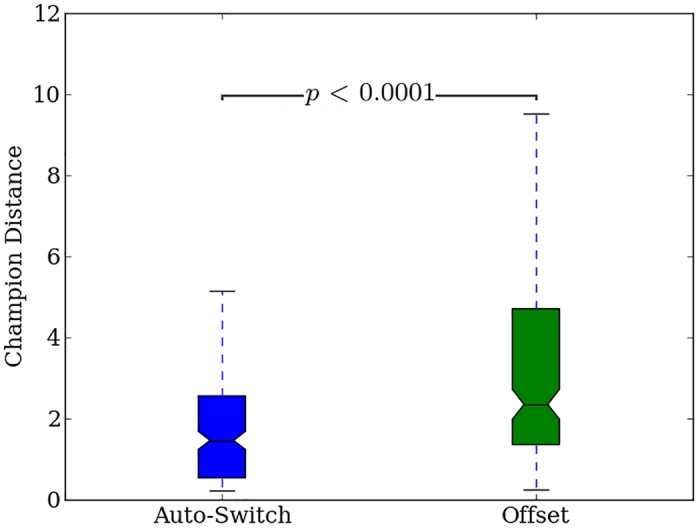
Final fitness for Offset-HybrID and Auto-Switch-HybrID on the quadruped robot transfer experiment. The distance traveled by Offset-HybrID and Auto-Switch-HybrID run champions after 3,000 generations averaged over all regularities of the quadruped robot transfer experiment.

Champion distance values for Offset-HybrID after 3,000 generations pooled over all regularities (*n* = 200) (2.34, [2.05, 2.9]) are significantly (*p* < 0.05) greater than Auto-Switch-HybrID (1.46, [1.05, 1.67]), showing that the indirect encoding is helpful when solving the regularities present in the new environment (Fig 24). The results of the transfer experiment confirm the hypothesis that Offset-HybrID’s concurrently-evolving genome allows for faster adaption to new environments through regular and coordinated mutations in the indirectly encoded component.

## Auto-Offset-HybrID

### Description

The switch-based HybrID methods seem to be capable of outperforming Offset-HybrID in many instances by evolving regularities during the pre-switch period without irregular mutations. To combine the strengths of both encodings, we have created Auto-Offset-HybrID, which automatically switches from evolving the CPPN genome to evolving directly encoded offsets while preserving the evolved CPPN. The method for determining the switch point is the same as Auto-Switch-HybrID (Eq 1). Like Offset-HybrID, the phenotype is produced by combining the CPPN output with the offsets. The design allows for evolution of regular phenotypes during the pre-switch period, after which offsets are evolved to address irregularity. Unlike Preset-Switch-HybrID and Auto-Switch-HybrID, the CPPN genome is preserved for future study and evolution. For all experiments, Auto-Offset-HybrID uses the same parameters for switching as Auto-Switch-HybrID and the same direct encoding mutation parameters as Offset-HybrID.

### Target weights experiment

**Fig 25:**
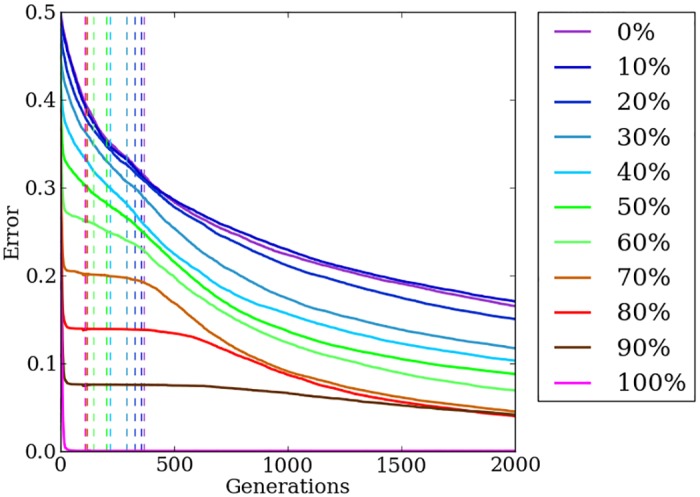
The mean error of Auto-Offset-HybrID generation champions over 2,000 generations on the target weights experiment. The line colors indicate the regularity of the problem tested. Dashed vertical lines indicate the mean switch point per treatment.

The behavior of Auto-Offset-HybrID before the switch point is identical to Auto-Switch-HybrID, as both evolve the CPPN genome only. Once the encoding switch is automatically triggered, Auto-Offset-HybrID evolves the directly encoded offsets to produce irregularity. Similar to Auto-Switch-HybrID, Auto-Offset-HybrID improves performance after the switch point for all problems with irregular elements (Fig 25), but at a slower rate than Auto-Switch-HybrID (Fig 6). The final error of Auto-Offset-HybrID run champions (*n* = 440) (0.0596, [0.0516, 0.084]) is significantly (*p* < 0.0001) greater than Auto-Switch-HybrID (0.0202, [0.0188, 0.0208]) and Preset-Switch-HybrID (0.0229, [0.0219, 0.0232]), but is significantly (*p* < 0.0001) less than HyperNEAT (0.2115, [0.1271, 0.2253]). Offset-HybrID still outperforms other encodings on the target weights problem (Fig 7).

Auto-Offset-HybrID treatments with less than 40% regularity produce larger (*p* > 0.004, NSABC) CPPN genomes (41, [36, 43]) than Offset-HybrID (27, [25, 28]) as the CPPN must evolve to solve irregularities without the directly encoded offsets before the switch point. On the other hand, Auto-Offset-HybrID treatments greater than 60% regular (16, [15, 17]) produce smaller (*p* > 0.004, NSABC) genomes than Offset-HybrID (23, [21, 24]), which provides more evidence that concurrently-evolving offsets interfere with the evolution of regularities.

### Bit mirroring experiment

The final fitness of Auto-Offset-HybrID run champions after 2,000 generations (*n* = 880) (304, [303, 304.01]) is significantly (*p* < 0.0001) greater than Offset-HybrID (301, [300.99, 301.01]), and HyperNEAT (302.98, [301.99, 303.04]) showing that the bit mirroring experiment, which exhibits epistasis, benefits from early evolution of regularity and later evolution of irregularities. The final fitness of Auto-Offset-HybrID champions (*n* = 880) is not significantly (*p* > 0.05) different from Auto-Switch-HybrID (305.01, [304.96, 306.081]) but is significantly (*p* < 0.05) less than Preset-Switch-HybrID (304.97, [303.94, 304.097]). Because all experiments are run with identical offset mutation settings, it is possible that the parameters might be fine-tuned to match the performance of Preset-Switch-HybrID.

### Quadruped robot experiment

**Fig 26:**
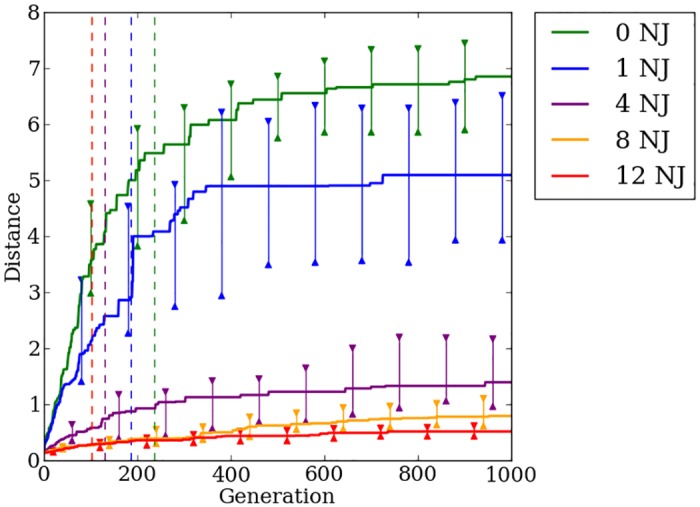
The median distance traveled by Auto-Offset-HybrID generation champions over 1,000 generations on the quadruped robot experiment. The line colors indicate the number of noisy joints on the robot. The median switch points per treatment are indicated by dashed lines. 95% bootstrapped confidence intervals of the median are shown every 100 generations.

**Fig 27:**
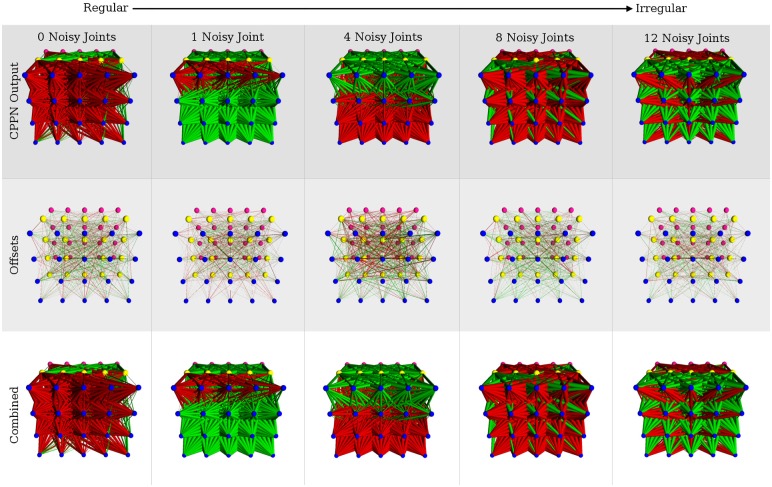
The fittest Auto-Offset-HybrID ANNs from each regularity of the quadruped robot experiment viewed from the output layer. Red links are inhibitory, green links are excitatory, and link weight magnitude is indicated by the thickness of the link.

Initial performance for Auto-Offset-HybrID on the quadruped robot experiment is similar to Auto-Switch-HybrID. Post-switch, Auto-Offset-HybrID performance improves somewhat slower than Auto-Switch-HybrID (Fig 26). Champion distance values for Auto-Offset-HybrID after 1,000 generations over all regularities (*n* = 200) (2.03, [1.34, 2.82]) are significantly (*p* < 0.05) less than Auto-Switch-HybrID (2.55, [2.25, 3.09]), but are significantly (*p* < 0.05) greater than Offset-HybrID (1.48, [1.07, 2.11]) and HyperNEAT (1.43, [0.91, 2.11]). The results show that the Auto-Offset-HybrID method effectively combines indirect and direct encodings, but that the direct encoding mutation parameters used by Auto-Switch-HybrID are more effective. The evolved Auto-Offset-HybrID quadruped ANN controllers appear visually similar to the evolved Offset-HybrID ANNs (Fig 27).

### Quadruped robot transfer experiment

**Fig 28:**
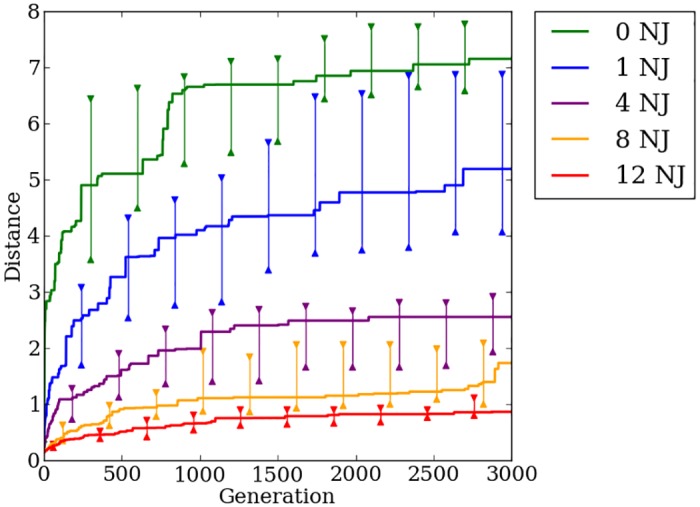
The median distance traveled by the transferred Auto-Offset-HybrID population generation champions over 3,000 generations on the quadruped robot transfer experiment. The line colors indicate the number of noisy joints on the robot. 95% bootstrapped confidence intervals of the median are shown every 300 generations.

For the quadruped robot transfer experiment, the Auto-Offset-HybrID population evolved for the previous quadruped robot problem is transferred to the new environment and evolved with the Offset-HybrID algorithm. This experiment shows the potential advantage of Auto-Offset-HybrID having the CPPN available post-switch, which is not true for Auto-Switch-HybrID. The transferred Auto-Offset-HybrID population quickly evolves to compensate for the new environment (Fig 28). Champion distance values for the transferred population after 3,000 generations pooled over all regularities (*n* = 200) (2.94, [2.4, 3.61]) are significantly (*p* < 0.0001) greater than Auto-Switch-HybrID (1.46, [1.05, 1.67]). However, no significant (*p* = 0.0658) difference in overall fitness exists between the transferred Auto-Offset-HybrID population and Offset-HybrID (2.34, [2.05, 2.9]).

We could have also tested evolving the Auto-Offset-HybrID (or Offset-HybrID) population on the transfer experiment by discarding the offset values, keeping the CPPNs, and restarting the Auto-Offset-HybrID (or Offset-HybrID) algorithm initialized with the transferred CPPNs, but where the new offsets evolve from scratch. However, because in this type of problem there are irregularities in the base environment that remain in the target environment, those methods would require evolution to re-solve the same irregularities that were solved in the pre-transfer experiment in addition to solving the regular change in the transfer environment. Nevertheless, such experiments would help identify the extent to which transferring the offsets are helpful, the degree to which simply having a CPPN in the target environment is helpful, and the degree to which the CPPN and the offsets have co-evolved in an intertwined way that affects their ability to be effectively transferred separately. Such experiments thus represent a fruitful opportunity for future research, and an opportunity to compare these new variants to the algorithms presented in this paper, including vanilla HyperNEAT. In the next section we outline other intriguing areas for future work.

## Future work

The new HybrID methods presented in this paper allow for many potentially interesting variations on their implementation. The effectiveness of automatic encoding switching might be improved by using different methods to measure fitness stagnation, or by allowing multiple switch points. The concurrently evolving genomes of Offset-HybrID allow for multiple implementations of crossover, such as performing crossover on the indirectly or directly encoded genome according to a probability value specified in the input parameters. Additionally, one could try co-evolving separate populations of direct and indirect encodings that get paired randomly (or in some other way) each generation. Due to limitations of time and space, we are unable to test all potential variations in this paper.

This paper demonstrates that indirect encodings handle irregularity most effectively when combined with a method for introducing directly encoded mutations. Learning algorithms directly manipulate ANN weights similar to the mutation of a direct encoding. One advantage of learning is that it is targeted, whereas mutation is random. The effectiveness of concurrently evolving direct and indirect encodings to address irregular problems might be reproduced or augmented by allowing learning to take place during fitness evaluation [42]. A key difference between Offset-HybrID and learning is that patterns within Offset-HybrID’s directly encoded genome are inherited by children while learned patterns are not usually passed on by a genome. To pass on learned patterns through the genome, a mechanism for epigenetic inheritance or communication of learning to offspring is required. Future work on addressing irregular problems may explore additional methods for combining learning and evolution.

## Conclusion

On the easiest problem, target weights, Offset-HybrID greatly outperforms all other encoding types, followed by Auto-Switch-HybrID, Preset-Switch-HybrID, Auto-Offset-HybrID and then HyperNEAT. HyperNEAT must use genome complexification to generate the irregular phenotypic structural patterns that are necessary to solve irregular problems, while Offset-HybrID is also capable of representing irregularities in the concurrently-evolving directly encoded genome. The directly encoded genome of Offset-HybrID becomes less-compressible as target weights problem irregularity increases, which shows that the offsets encode problem irregularities.

On the bit mirroring problem, Auto-Switch-HybrID, Preset-Switch-HybrID, and Auto-Offset-HybrID outperform Offset-HybrID and HyperNEAT. The switch-based encodings are able to fine-tune performance with the direct encoding, while concurrently evolving encodings produces little benefit. Comparing the size of indirectly encoded genomes evolved with HyperNEAT and Offset-HybrID shows that the concurrently-evolved offsets are unable to encode the bit mirroring problem irregularities as effectively as the target weights irregularities.

Preset-Switch-HybrID, Auto-Switch-HybrID, and Auto-Offset-HybrID also outperform HyperNEAT and Offset-HybrID on the quadruped problem. Auto-Switch-HybrID is able to successfully automate the encoding switch point while performing as well as Preset-Switch-HybrID on the quadruped robot and bit mirroring experiments, while outperforming Preset-Switch-HybrID on the target weights experiment. The successful tests verify that automatically switching from indirect to direct encoding is useful for solving irregular problems.

It is interesting that Offset-HybrID is least-effective when fitness is determined by the output of the phenotype, such as the bit mirroring and quadruped experiments, instead of the evolved topology, as in the target weights experiment. The differences in performance could be due to the increased difficulty of the bit mirroring and quadruped robot experiments resulting from epistasis. Preset-Switch-HybrID, Auto-Switch-HybrID, and Auto-Offset-HybrID show that a direct encoding is able to optimize solutions on problems with epistasis after an indirect encoding solves problem regularities.

The concurrent evolution of indirect and direct encodings can be preferable to switching from an indirect encoding to a direct encoding in situations where populations are to be transferred between related tasks. The indirectly encoded genome allows for faster, large-scale, regular changes in the phenotype via mutation while a directly encoded genome can only be altered by slower small-scale fine-tuning that is not suited to produce the types of mutations needed to solve the transfer problem. The most-regular treatments of the quadruped transfer experiment show that Offset-HybrID accounts for regular changes to the problem through coordinated mutation effects produced by the indirectly encoded genome. Auto-Switch-HybrID (post-encoding-switch) is unable to take advantage of regularity in the changes to the problem and is ineffective on regular treatments compared to Offset-HybrID.

Our work has shown that Offset-HybrID and Auto-Switch-HybrID are capable of out-performing HyperNEAT to evolve solutions for certain irregular problems. Auto-Switch-HybrID improves on Preset-Switch-HybrID to reduce the level of human intervention needed for switch-based HybrID methods, while Offset-HybrID quickly solves simple irregular problems and evolves to account for regular changes in the problem description. Work with Offset-HybrID also suggests possible designs for more effectively combining encoding methods. Overall, the encoding methods developed for this paper are effective tools for automatically generating solutions to difficult engineering problems.
